# Doping Lanthanide Nanocrystals With Non-lanthanide Ions to Simultaneously Enhance Up- and Down-Conversion Luminescence

**DOI:** 10.3389/fchem.2020.00832

**Published:** 2020-09-23

**Authors:** Yingying Li, Chunyan Liu, Peisen Zhang, Jiayi Huang, Haoran Ning, Peng Xiao, Yi Hou, Lihong Jing, Mingyuan Gao

**Affiliations:** ^1^Key Laboratory of Colloid, Interface and Chemical Thermodynamics, Institute of Chemistry, Chinese Academy of Sciences, Beijing, China; ^2^School of Chemistry and Chemical Engineering, University of Chinese Academy of Sciences, Beijing, China; ^3^Center for Molecular Imaging and Nuclear Medicine, School for Radiological and Interdisciplinary Sciences (RAD-X), Collaborative Innovation Center of Radiation Medicine of Jiangsu Higher Education Institutions, and State Key Laboratory of Radiation Medicine and Protection, Soochow University, Suzhou, China

**Keywords:** non-lanthanide doping, up- and down-conversion luminescence, crystal field, valence state effects, rare-earth nanocrystals, lanthanide emitters

## Abstract

The rare-earth nanocrystals containing Er^3+^ emitters offer very promising tools for imaging applications, as they can not only exhibit up-conversion luminescence but also down-conversion luminescence in the second near-infrared window (NIR II). Doping non-lanthanide cations into host matrix was demonstrated to be an effective measure for improving the luminescence efficiency of Er^3+^ ions, while still awaiting in-depth investigations on the effects of dopants especially those with high valence states on the optical properties of lanthanide nanocrystals. To address this issue, tetravalent Zr^4+^ doped hexagonal NaGdF_4_:Yb,Er nanocrystals were prepared, and the enhancement effects of the Zr^4+^ doping level on both up-conversion luminescence in the visible window and down-conversion luminescence in NIR II window were investigated, with steady-state and transient luminescence spectroscopies. The key role of the local crystal field distortions around Er^3+^ emitters was elucidated in combination with the results based on both of Zr^4+^ and its lower valence counterparts, e.g., Sc^3+^, Mg^2+^, Mn^2+^. Univalent ions such as Li^+^ was utilized to substitute Na^+^ ion rather than Gd^3+^, and the synergistic effects of Zr^4+^ and Li^+^ ions by co-doping them into NaGdF_4_:Yb,Er nanocrystals were investigated toward optimal enhancement. Upon optimization, the up-conversion emission of co-doped NaGdF_4_:Yb,Er nanocrystals was enhanced by more than one order of magnitude compared with undoped nanocrystals. The current studies thus demonstrate that the local crystal field surrounding emitters is an effective parameter for manipulating the luminescence of lanthanide emitters.

## Introduction

Rare-earth (RE) nanocrystals are promising light-emitting materials as they are able to convert near infrared (NIR) photons into ultraviolet, visible, and NIR lights through either an up-conversion path or a down-conversion path (Chen et al., 2013; Dong et al., 2015). Compared with conventional down-conversion luminescent materials such as organic dyes and semiconductor quantum dots (Antaris et al., 2016; Chen B.K. et al., 2017; Stroyuk et al., 2018; Lu et al., 2020), rare-earth nanocrystals offer remarkable optical advantages, such as narrow “atomic-line” emission from the internal f-f transitions of lanthanide ions, large stokes or anti-stokes shifts, long luminescence lifetime, and high photochemical stability, and thus are potentially useful for diverse imaging applications (Zhou et al., 2012; Liu et al., 2014; Fan et al., 2018; Ai et al., 2019). Especially, emitting lanthanide Er^3+^ ions based rare-earth nanocrystals have aroused intense interests because Er^3+^ ions present a longer down-conversion emission wavelength at 1,525 nm in the second NIR window (NIR II, 1,000–1,700 nm), which enables a virtual zero auto-fluorescence interference of tissues in bio-imaging, apart from the up-conversion emission at 541 nm and 656 nm in the visible and NIR I region (NIR I, 650–950 nm), respectively (Shen et al., 2013; Xu et al., 2019). However, due to the low absorption cross-section (~10^−20^ cm^2^) of lanthanide ions caused by the parity-forbidden transition of 4f electrons (Tu et al., 2015), and non-radiative relaxation between multi-energy levels, their up- and down-conversion luminescence efficiencies are generally low, which obviously hinders their applications. In principle, Yb^3+^ ions are suitable for sensitizing the up-conversion luminescence of Er^3+^ ions, benefiting from the large absorption cross-section of Yb^3+^ ions. Increasing the concentration of sensitizer or emitter have been adopted to improve the luminescence upon 980 nm excitation (Chen Q.S. et al., 2017; Ma et al., 2017), but overincreasing the concentration of Yb^3+^ is unfavorable owing to the participation of Yb^3+^ ions on the particle surface in dissipating the absorbed energy (Johnson et al., 2017).

Apart from sensitizing ion doping, significant progresses have also been made for improving the up-conversion luminescence of Er^3+^ ions in rare-earth nanocrystals through surface passivation by forming core-shell structures (Yi and Chow, 2007; Fan et al., 2019), surface plasmon coupling by conjugating to noble metal particles (Sun et al., 2014; Clarke et al., 2018), and host-lattice manipulation by non-lanthanide (Ln) ions doping (Dong et al., 2013). The epitaxial growth of a shell on preformed emitting core, e.g., growing NaGdF_4_ shell on NaGdF_4_:Yb,Er core (Vetrone et al., 2009; Li et al., 2013), can suppress the energy transfer from the emitting core to surrounding environment, thus favorable for boosting the luminescence efficiency, apart from giving rise to stable and controllable core/shell structures (Wang et al., 2010). However, this approach will require at least two successive steps of preparations (He et al., 2017). Modifying rare-earth nanocrystals with plasmonic noble metal can efficiently improve the luminescence by changing the surface electronic field, but also leads to relatively large and less stable structures that may be disassociated under certain conditions. Different from these two approaches, doping non-Ln ions to enhance the luminescence through modulating the local chemical environment of the emitting centers doesn't necessarily increase the particle size apart from forming more stable structures (Huang et al., 2019). Because of the interplay between the 4f electrons of Er^3+^ and the crystal field of the host doped with non-Ln ions, the probability of radiative transitions within Er^3+^ can hopefully be enhanced by relaxing the selection rules (Fischer et al., 2019). For example, metal ions such as Li^+^, Ca^2+^, Mn^2+^, and Fe^3+^, were adopted as non-Ln dopants to enhance the up-conversion luminescence (Cheng et al., 2012; Zeng et al., 2014; Tang et al., 2020b; Verma et al., 2020). Doping of Li^+^ ions was demonstrated to increase the up-conversion luminescence of Y_2_O_3_:Yb,Er nanocrystals by factors of 8-25 (Chen et al., 2008). Doping of alkaline-earth ions such as Ca^2+^ was also found to improve the uniformity of the resulting NaGdF_4_:Yb,Er nanocrystals, apart from enhancing the up-conversion luminescence (Lei et al., 2013). Doping Fe^3+^ into a NaYF_4_:Yb,Er nanocrystals selectively enhanced the red up-conversion luminescence of Er^3+^ ions by 7 times (Tang et al., 2015). Nevertheless, doping of 3*d* transition metal ions such as Mn^2+^ or Fe^3+^ into host will introduce new energy levels associated with partially filled 3*d* orbitals of the dopant, particularly when the doping level is high enough. In consequence, additional electronic transition pathways may be created to alter the up-conversion emission position or decrease the up-conversion luminescence intensity. However, the effect of dopant ions on the down-conversion luminescence of Er^3+^ remains rarely addressed. Besides, the dopants are mainly chosen from low valence state ions. Therefore, it is fundamentally interesting to study the high valence dopants particularly those free of d*-*d transitions for improving the up- and down-conversion luminescence of Er^3+^ based RE luminescent nanocrystals. But there is a clear lack of a systematic study in this respect.

Following our previous investigations on the synthesis and optical properties of RE nanocrystals (Liu et al., 2013; Huang et al., 2015; Li et al., 2019), herein we report hexagonal NaGdF_4_:Yb,Er nanocrystals doped with tetravalent Zr^4+^ ions that possess d^0^ configuration, and studied the Zr^4+^ doping level-dependent luminescence properties of Er^3+^ through both steady-state and transient luminescence spectroscopies. The resulting Zr^4+^-doped NaGdF_4_:Yb,Er nanocrystals exhibited enhanced emissions for both up-conversion luminescence and down-conversion emission peaking at 1,525 nm. The role of Zr^4+^ dopants in altering the local crystal field of the emitters was discussed in combination with Judd-Ofelt theory. To disclose the underlying mechanisms, the effects of the valence state of the dopant ions on the luminescence properties were investigated by doping NaGdF_4_:Yb,Er nanocrystals with Sc^3+^, Mg^2+^, Mn^2+^, respectively, for comparing with the Zr^4+^-doped counterpart. Different from above dopants, univalent ion such as Li^+^ was doped into NaGdF_4_:Yb,Er nanocrystals by substituting Na^+^ rather than Gd^3+^, and the synergistic effects of Zr^4+^ and Li^+^ ions by co-doping them into NaGdF_4_:Yb,Er nanocrystals were investigated toward optimal enhancement.

## Results and Discussion

### Synthesis of Zr^4+^-Doped NaGdF_4_:Yb,Er Nanocrystals

**Figure 1:**
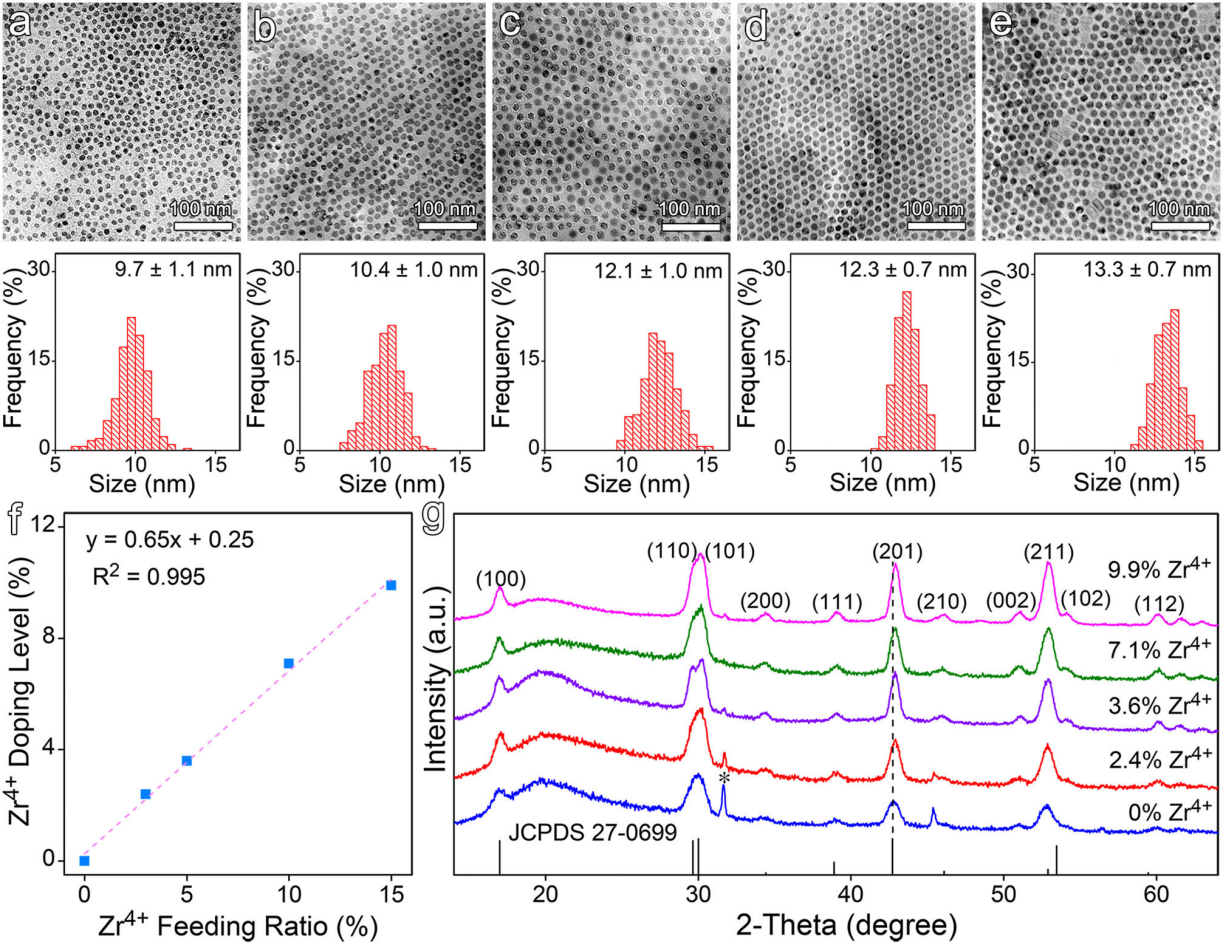
**(a–e)** Typical TEM images of NaGdF_4_:Yb,Er nanocrystals doped with different feeding ratios of Zr^4+^ ions, i.e., **(a)** 0, **(b)** 3.0%, **(c)** 5.0%, **(d)** 10.0%, and **(e)** 15.0%, together with their corresponding size distributions, respectively. **(f)** The actual doping levels of Zr^4+^ in the final nanocrystals. **(g)** The XRD patterns of NaGdF_4_:Yb,Er nanocrystals before and after Zr^4+^ doping. The vertical line pattern in the bottom frame is the standard XRD data for hexagonal NaGdF_4_ according to JCPDS card.

Zr^4+^-doped NaGdF_4_:Yb,Er nanocrystals were synthesized via a high temperature approach using oleic acid (OA) as the particle surface capping ligand. As displayed in Figure 1a, the as-prepared NaGdF_4_:Yb,Er nanocrystals are quasi-spherical. After doped with Zr^4+^ ions, as shown in Figures 1b–e, the nanocrystals slightly increase in particle size from 9.7 ± 1.1 nm to 13.3 ± 0.7 nm, depending on the initial feeding molar ratio of Zr^4+^ to the total cations. Meanwhile, the relative standard deviation (RSD) of the particle size was reduced from 11.3 to 5.3%. Further ICP-AES results demonstrated that the Zr^4+^ ions were successfully doped into the NaGdF_4_:Yb,Er nanocrystals and the actual doping level of Zr^4+^ in the final particles goes linearly from 2.4 to 9.9% against the initial feeding molar ratio of Zr^4+^ from 3.0 to 15.0%, as shown in Figure 1f.

It's worth noting that the size of the doped nanocrystals is positively correlated with the doping level of Zr^4+^ ions. In the current reaction system, OA acts as a coordinating solvent. It forms metal oleate with the metal precursors prior to the formation of the target nanocrystals. In this context, OA is expected to show higher binding affinity to Zr^4+^ ion than to Gd^3+^ ion due to the smaller radius and higher valence of Zr^4+^. The energy barrier for decomposing Zr oleate is expected to be higher than that for Gd oleate, which can find supports from the difference in the bond dissociation energies, i.e., 766.1 kJ/mol for Zr-O and 715 kJ/mol for Gd-O. Consequently, the supersaturation level as well as the number of nuclei is decreased in the reaction system as a function of Zr^4+^ precursor concentration, which explains the dependency of particle size on the Zr^4+^ feeding ratio (Buonsanti and Milliron, 2013).

The crystal structure of all aforementioned samples was analyzed by powder XRD. As shown in Figure 1g, both undoped and Zr^4+^-doped NaGdF_4_:Yb,Er nanocrystals display a hexagonal phase structure. Although the doped samples exhibit similar diffraction patterns, the diffraction peak of (201) crystal plane gradually shifts to higher angles along with the increase of Zr^4+^ dopant concentration, indicating an effective incorporation of Zr^4+^ ions into the host lattice. It can then be deduced that the Zr^4+^ ions substitute Gd^3+^ ions rather than staying at the interstitial sites, because substituting the Gd^3+^ cations with smaller Zr^4+^ ions will cause the host lattice to shrink according to Bragg's Law (2dsinθ = nλ), while occupying the interstitial sites will cause the host lattice to expand. According to the ICP-AES results given in Table S1 in supplementary material the concentration of Gd^3+^ in the nanocrystals was decreased with the increase of Zr^4+^ doping level, which strongly supports the above structural analysis. While the gradually narrowed full width at half maximum (FWHM) of the main diffraction peaks, as shown in Figure S1, suggested that Zr^4+^ doping didn't decrease the crystalline degree, which is the most important prerequisite for improving the luminescence properties of the doped nanocrystals through the manipulation of the crystal field around the emitting ions.

### Up- and Down-Conversion Luminescence of Zr^4+^-Doped NaGdF_4_:Yb,Er Nanocrystals

**Figure 2:**
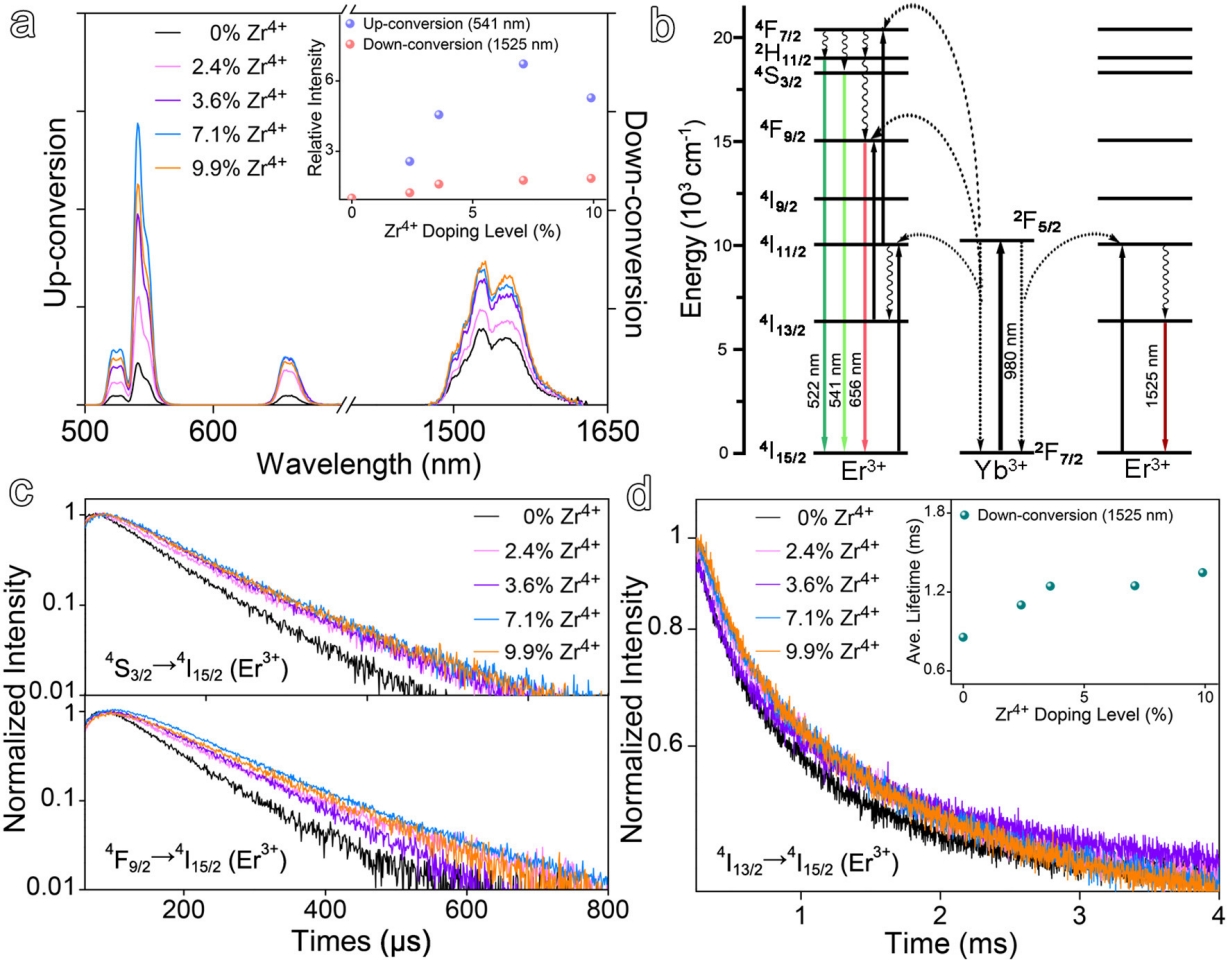
**(a)** Up- and down-conversion luminescence spectra of Zr^4+^-doped NaGdF_4_:Yb,Er nanocrystals at varying doping ratios of Zr^4+^ ions under 980 nm laser excitation and the up- and down-conversion emission intensities as a function of Zr^4+^ doping level, respectively. **(b)** Simplified energy level diagrams depicting the energy transfer between Yb^3+^ and Er^3+^ ions. **(c,d)** Transient emissions observed at 541, 656, and 1,525 nm of NaGdF_4_:Yb,Er nanocrystals at varying doping ratios of Zr^4+^ ions.

As demonstrated by the results given in Figure 1 that the particle size and size distribution are dependent on the doping level of Zr^4+^, it is interesting to learn the dependency of optical properties of the resulting nanocrystals on the doping level of Zr^4+^ as well. In the absence of Zr^4+^ dopant, the NaGdF_4_:Yb,Er nanocrystals exhibit multiple emissions upon 980 nm excitation e.g., up-conversion emissions peaking at 541 and 656 nm, down-conversion emission peaking at 1,525 nm, respectively, as shown in Figure 2a. In the presence of Zr^4+^ dopants, the luminescence intensity of the up-conversion emission at 541 nm is dramatically increased against the Zr^4+^ content and gets enhanced by a factor of 6.7 when the Zr^4+^ content reaches 7.1%, and then becomes decreased upon further increase of the Zr^4+^ content. Meanwhile, the luminescence intensity of the down-conversion emission presents a similar tendency and gets enhanced by a factor of 1.8 when the Zr^4+^ content is around 7.1%.

To rule out the possible size effects on the optical properties, the undoped NaGdF_4_:Yb,Er nanocrystals of 13.2 nm were prepared, as shown in Figure S2a, for comparing with the Zr^4+^-doped NaGdF_4_:Yb,Er with Zr^4+^ level of 9.9%. The enhancement factors for up-conversion emission at 541 nm and down-conversion emission at 1,525 nm were determined to be 1.2 and 1.1, respectively, as shown in Figure S2b, remarkably smaller than that obtained upon Zr^4+^ doping. Therefore, it is reasonable to deduce that the enhancement effects on both up- and down-conversion luminescence may come from the alternation of crystal field symmetry surrounding Er^3+^ ions, induced by the Zr^4+^ dopants. Regarding the difference in the enhancement effects of Zr^4+^ ions on up- and down-conversion emissions, it can reasonably be attributed to the difference between the transitions involved in these two types emissions, as shown in Figure 2b. The up-conversion luminescence of Er^3+^ is a multiphoton process and requires the participation of multiple energy levels, while the down-conversion emission of Er^3+^ involves less energy levels. Therefore, it can be expected that the up-conversion luminescence is more readily disturbed by the crystal field.

To provide more evidence on the variation of the crystal field induced by Zr^4+^ ions, time-resolved luminescence measurements were carried out to determine the variations in the lifetimes of up- and down-conversion luminescence upon Zr^4+^ doping. The luminescence decay curves measured at different emission positions, i.e., 541 nm (^4^S_3/2_-^4^I_15/2_), 656 nm (^4^F_9/2_-^4^I_15/2_), and 1,525 nm (^4^I_13/2_-^4^I_15/2_) of the Zr^4+^-doped NaGdF_4_:Yb,Er nanocrystals with different Zr^4+^ doping levels are shown in Figures 2c,d. All decay curves were well-fitted with a two-exponential function and the detailed fitting results are given in Table S2. In brief, Zr^4+^ doping increased the average lifetime of 541 nm emission from 82.1 μs for the undoped ones to 114.1 μs for doped ones with Zr^4+^ content of 7.1%, while the average lifetime of 1,525 nm emission was prolonged from 855.0 μs to 1247.2 μs accordingly. The measured lifetime of the excited states of Er^3+^ is the reciprocal of the total transition rate including radiative and non-radiative transition rates. The non-radiative transition rate of Er^3+^ can be affected by the phonon energy of NaGdF_4_ lattice, the energy transfer process from adsorbed photoenergy to surroundings, the cross-relaxation between energy levels, and so forth. While the radiative transition part strongly depends on the crystal structure and the symmetry of local crystal field of the emitters owing to a decrease of the forbidden nature of the transition in asymmetric sites (Boyer et al., 2004; Tu et al., 2013). In general, the lifetime of the intermediate states of activator experiences a prolongation with the distortion of its local asymmetry (Kar and Patra, 2012; Dong et al., 2015). Therefore, the prolongation of the lifetimes of both up- and down-conversion luminescence suggests that long-lived intermediate excited states ^4^I_11/2_ and the population of ^4^S_3/2_ and ^4^I_13/2_ (Er^3+^) become more favorable upon the introduction of Zr^4+^ dopants, which as a result increases the f-f transition probability of Er^3+^ to induce efficient up- and down-conversion luminescence.

Trivalent lanthanide ions generally suffer from low luminescence efficiency as the electric-dipole transition within the 4f electrons is forbidden by Laporte rule. The non-Ln ions doping may disturb the local crystal field of the emitters and thus change the transition probability (Chen et al., 2014; Han et al., 2014). In order to further understand the underlying mechanism of the Zr^4+^ dopants governing the luminescence, the electric-dipole transition theory was discussed below.

The f-f transition intensity can be estimated from Judd-Ofelt theory as given by Equations (1) and (2) (Werts et al., 2002; Du et al., 2018),

(1) $$\begin{array}{r} A=\;\frac{64\pi^{4}e^{2}}{3h\left(2J+1\right)\lambda^{3}}\left[\frac{{n\left(n^{2}+2\right)}^{2}}{9}S_{ed}+n^{3}S_{md}\right] \end{array}$$

(2) $$\begin{array}{r} S_{ed}=\;\sum_{t=2,\;4,\;6}\Omega_{t}|\langle f^{n}\;\left[S,\;L\right]J||U^{t}||f^{n}\left[S^{\prime },L^{\prime }\right]\;J^{\prime }\rangle {|}^{2} \end{array}$$

where *A* is the spontaneous emission probability of transition from the initial *J* manifold |[*S, L*]*J*〉 to final *J*′ manifold |[*S*′, *L*′]*J*′〉 and *e, h, n*, and λ refer to the elementary charge, Plank constant, refraction index, and average wavelength of the transition, respectively. 2*J*+1 is the degeneracy of the initial state, and *S*_ed_ and *S*_md_ are the electric dipole and magnetic dipole line strengths, respectively. The electric dipole transition strength *S*_ed_ is very sensitive to the chemical surrounding of the lanthanide emitting ions, whereas the magnetic dipole transition strength *S*_md_ is not. In Equation (2), *S*_ed_ can be calculated by three transition intensity parameters Ω_*t*_ (*t* = 2, 4, 6). Ω_2_ is the spectral intensity parameter of electric-dipole transition for 4f^n^ electron configuration, strongly depending on the crystal field environment and reflecting that the asymmetry of the crystal field surrounding lanthanide emitting ions (Zhang et al., 2018). In principle, the enhanced lattice distortion leads to lower local crystal field symmetry, thereby giving rise to larger value of Ω_2_ (Walsh et al., 1998; Liu et al., 2008). With relatively smaller cationic radius and higher valence state of Zr^4+^ compared with those of Gd^3+^, the bond length of Zr-F (1.90 Å) is shorter than that of Gd-F (1.99 Å), whilst the bond energy of Zr-F (627.2 kJ/mol) is higher than that of Gd-F (590 kJ/mol), indicating that Zr^4+^ ions have stronger electron cloud distortion ability than Gd^3+^ ions. This can be further evidenced by a stronger electronic polarizability of Zr^4+^ (3.56 × 10^−24^ cm^3^) than that of Gd^3+^ (3.39 × 10^−24^ cm^3^) (Shannon and Fischer, 2016). Therefore, the distortion of electron clouds of Er^3+^ ions becomes favorable upon substitution of Gd^3+^ with Zr^4+^ dopants, which is intensified against the Zr^4+^ doping level. The decreased local crystal field symmetry is expected to give rise to a higher Ω_2_, which consequently encourages the electronic coupling between 4f energy levels and increases the probability of the intra f-f transition, leading to more effective enhancement in up-conversion emission.

### NaGdF_4_:Yb,Er Nanocrytals Doped With Cations of Various Valance States

To further show the effects of the valence state of cationic dopants on the emission of Er^3+^ ions via the modulation of the local crystal field, non-Ln cations with various valance states, e.g., Sc^3+^, Mg^2+^, Mn^2+^, were also chosen to dope the NaGdF_4_:Yb,Er nanocrystals by feeding molar ratio of 10%, according to that optimized for Zr^4+^.

**Figure 3:**
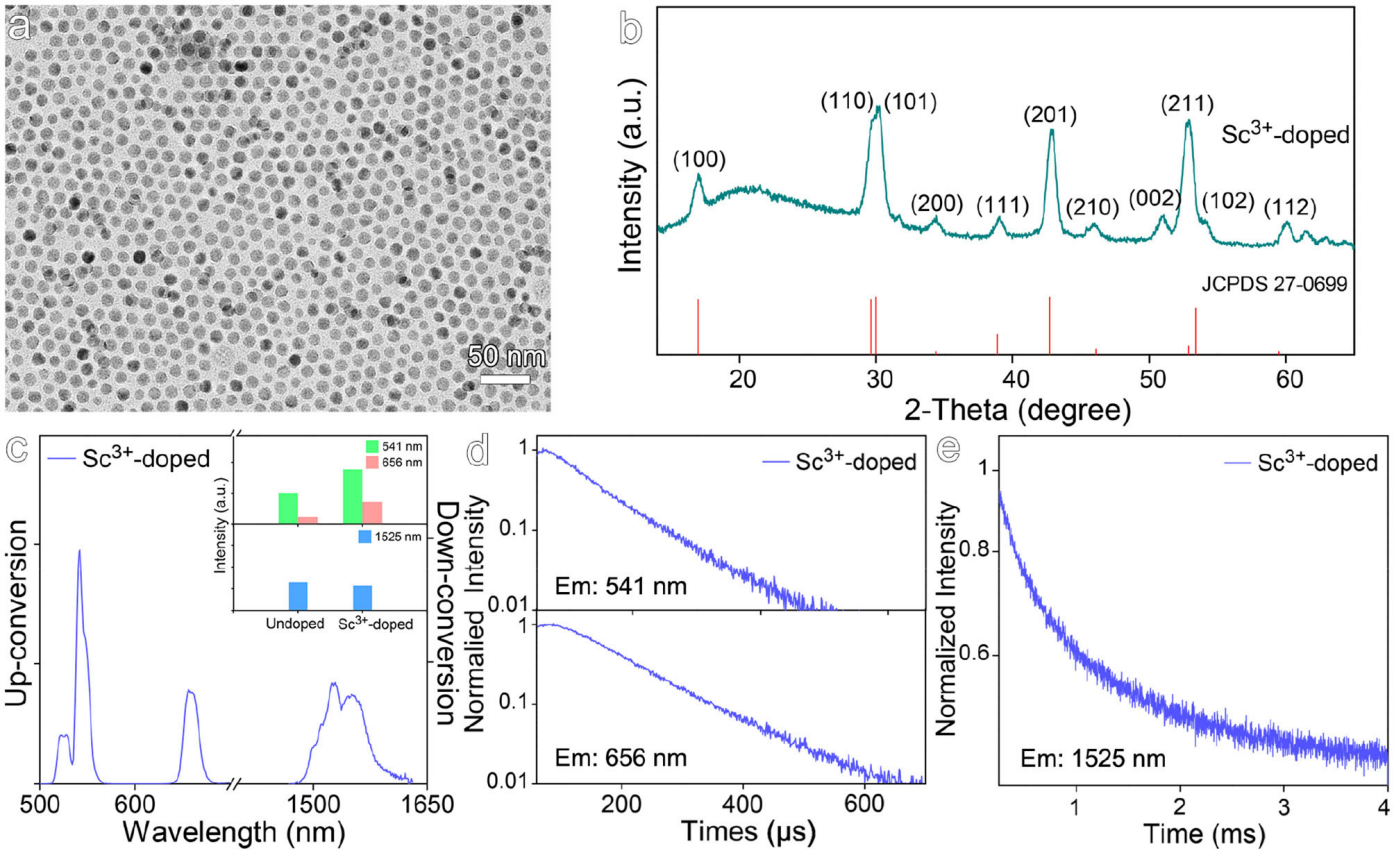
**(a)** TEM image and **(b)** corresponding XRD pattern of Sc^3+^-doped NaGdF_4_:Yb,Er nanocrystals. **(c)** The up- and down-conversion luminescence spectra and intensities at 541, 656, and 1,525 nm of Sc^3+^-doped NaGdF_4_:Yb,Er nanocrystals under 980 nm laser excitation. **(d,e)** Transient emissions observed at 541, 656, and 1,525 nm of Sc^3+^-doped NaGdF_4_:Yb,Er nanocrystal.

Although Sc^3+^ ion has the same valence as Gd^3+^ ion, it was selected to dope NaGdF_4_:Yb,Er by substituting Gd^3+^ ion. As it possesses no electron in *d* orbital, the substitution won't introduce new energetic levels into the nanocrystals. As shown in Figure 3a, the particle size of the resulting Sc^3+^-doped nanocrystals is 12.6 ± 0.8 nm. The XRD pattern shown in Figure 3b reveals the nanocrystals are in hexagonal phase. According to the luminescence spectra given in Figure 3c, Sc^3+^ doping only slightly increases the up-conversion luminescence by a factor of 1.8 and shows almost no impact on the down-conversion luminescence. As shown in Figures 3d,e and Table S3, **S**c^3+^ doping gives rise to a slight increase in the average lifetimes of 541 nm and 656 nm emissions, i.e., from 82.1 μs to 85.9 μs and from 98.7 μs to 118.1 μs, respectively. The average lifetime of down-conversion emission at 1,525 nm was increased from 855.0 μs to 1035.9 μs, but still lower than that for Zr^4+^-doped counterparts. This can be understood by the fact that Sc^3+^ has very similar properties to Gd^3+^ as seen by very comparable binding energies between Sc-F (599 kJ/mol) and Gd-F (590 kJ/mol) bonds, thus it won't introduce strong distortion effects on the crystal field surrounding Er^3+^ as Zr^4+^ does.

**Figure 4:**
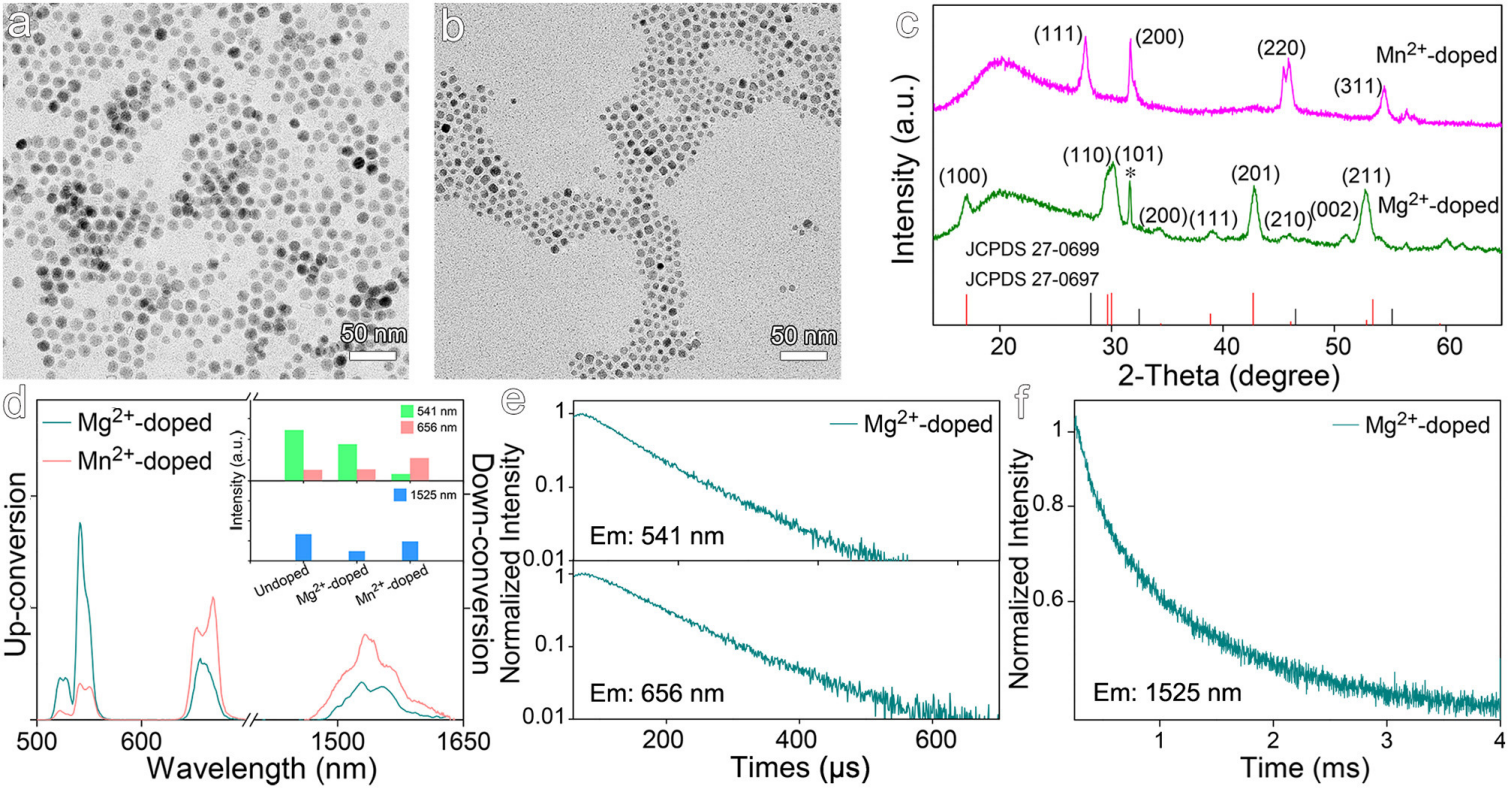
TEM images of NaGdF_4_:Yb,Er nanocrystals doped with **(a)** Mg^2+^ and **(b)** Mn^2+^. **(c)** Corresponding XRD patterns of NaGdF_4_:Yb,Er nanocrystals doped with Mg^2+^ and Mn^2+^. The vertical line pattern in the bottom frame is the standard XRD data for hexagonal (red line) and cubic (black line) NaGdF_4_ according to JCPDS card. **(d)** The up- and down-conversion luminescence spectra and intensities at 541, 656, and 1,525 nm of NaGdF_4_:Yb,Er nanocrystals doped with Mg^2+^ and Mn^2+^ under 980 nm laser excitation. **(e,f)** Transient emissions observed at 541, 656, and 1,525 nm of Mg^2+^-doped NaGdF_4_:Yb,Er nanocrystal.

Both Mg^2+^ and Mn^2+^ were selected to dope NaGdF_4_:Yb,Er nanocrystals, because the former is free of *d* orbital electrons contrasting to the latter that has *d*^5^ electron configuration. Therefore, it is expected to show the impacts of electronic configuration of the dopants on the optical properties of the resulting nanocrystals. As shown in Figures 4a,b, the particle sizes of the resulting Mg^2+^-doped and Mn^2+^-doped nanocrystals are of 12.4 ± 1.0 nm and 7.2 ± 1.0 nm, respectively. The XRD results shown in Figure 4c reveal that Mg^2+^-doped nanocrystals display a hexagonal phase structure, while Mn^2+^-doped nanocrystals exhibit a distinct cubic phase structure. The results in Figure 4d further reveal that Mg^2+^ doping slightly decreases the emission at 541 nm and the emission at 1,525 nm, while the emission intensity at 656 nm remains nearly unchanged. This can be explained by the fact that Mg^2+^ ions have much weaker electron cloud distortion ability than Gd^3+^ due to low electric polarizability (0.6 × 10^−24^ cm^3^), and thereby Mg^2+^-doped nanocrystals display even weakened luminescence in comparison with the undoped NaGdF_4_:Yb,Er nanocrystals. As shown in Figures 4e,f and Table S3, Mg^2+^ dopants give rise to a slightly increased average lifetimes for 541 nm and 656 nm emissions, i.e., from 82.1 μs to 87.4 μs and 98.7 μs to 115.4 μs in comparison with the undoped one. The average lifetime of down-conversion emission at 1,525 nm was increased from 855.0 μs to 962.8 μs, even shorter than that of Sc^3+^-doped nanocrystals let alone the lifetime of Zr^4+^-doped ones. Therefore, it can be concluded that Mg^2+^ ions can hardly distort the crystal filed surrounding Er^3+^ ions. Moreover, the substitution of Ln^3+^ ions with bivalent Mg^2+^ ions in the NaGdF_4_:Yb,Er nanocrystals will generate F^−^ vacancies to balance the charge of the whole nanocrystal, which is even deleterious to the luminescence as the increase of the concentration of defects around the emitters (Tang et al., 2020a).

When it comes to Mn^2+^ dopants, the intensity of 541 nm emission is dramatically decreased by a factor of 7.5, while the intensity of 656 nm emission is increased by a factor of 2.1, in comparison with those recorded from undoped counterparts. Consequently, the ratio of red emission to green emission is dramatically increased as shown in Figure 4d, arising from the involvement of *d* electron-associated energy levels of Mn^2+^ ions. It has been reported that using KMnF_3_ as matrix, a single-band red emission was obtained upon Mn-doping (Wang et al., 2011). Although the intensity of 1,525 nm emission is nearly unchanged in comparison with that of undoped nanocrystals, the down-conversion emission profile of Er^3+^ ions is also varied upon Mn^2+^ doping.

### NaGdF_4_:Yb,Er Nanocrystals Doped With Univalent Ions

**Figure 5:**
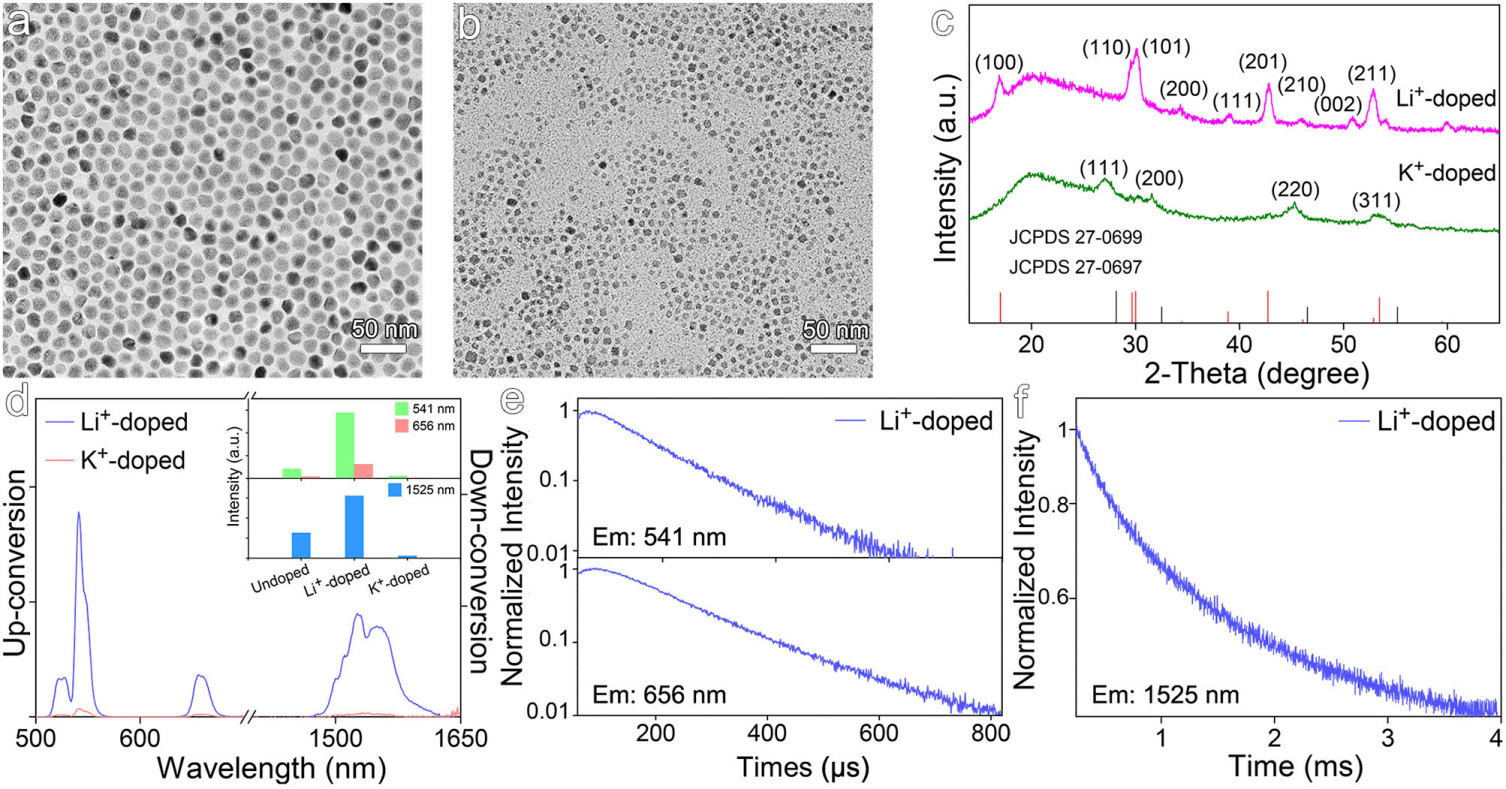
TEM images of NaGdF_4_:Yb,Er nanocrystals doped with **(a)** Li^+^ and **(b)** K^+^. **(c)** Corresponding XRD patterns of NaGdF_4_:Yb,Er nanocrystals doped with Li^+^ and K^+^. **(d)** The up- and down-conversion luminescence spectra and intensities at 541, 656, and 1,525 nm of NaGdF_4_:Yb,Er nanocrystals doped with Li^+^ and K^+^ under 980 nm laser excitation, respectively. **(e,f)** Transient emissions observed at 541, 656, and 1,525 nm of Li^+^-doped NaGdF_4_:Yb,Er nanocrystal.

Different from the above-mentioned mechanism for enhancing the luminescence of Er^3+^ emitters through the distortion of local crystal field, univalent ions such as Li^+^ and K^+^ were also used to dope NaGdF_4_:Yb,Er nanocrystals by substituting Na^+^ rather than Gd^3+^. The TEM results shown in Figures 5a,b reveal that the average size of Li^+^-doped nanocrystals is of 14.3 ± 1.2 nm, while that of K^+^-doped nanocrystals becomes greatly decreased to 6.8 ± 1.0 nm. The XRD results in Figure 5c reveal that Li^+^-doped nanocrystals are in hexagonal phase, but K^+^-doped nanocrystals are in cubic phase. As the diffraction peak of (201) crystal plane of Li^+^-doped NaGdF_4_:Yb,Er shifts higher angles, it can be deduced that Li^+^ ions take the positions of Na^+^ ions rather than occupy the interstitial sites. According to luminescence spectra of the Li^+^- and K^+^-doped NaGdF_4_:Yb,Er nanocrystals shown in Figure 5d, Li^+^ doping increases the intensities of 541 nm emission and 1,525 nm emission by factors of 6.8 and 2.5, respectively. In huge contrast, the up- and down-conversion luminescence of K^+^-doped nanocrystals are dramatically decreased, due to the remarkably decreased particle size and higher crystal field symmetry of cubic phase. As shown in Figures 5e,f and Table S4, Li^+^ doping significantly prolongs the average lifetimes of emissions at 541 and 656 nm, i.e., from 82.1 μs to 107.5 μs, and from 98.7 μs to 151.1 μs, respectively. Meanwhile, Li^+^ doping also profoundly prolongs the average lifetime of the emission at 1,525 nm, i.e., from 855.0 μs to 1297.5 μs, which is comparable with 1247.2 μs for Zr^4+^-doped ones. In fact, owing to the much smaller radius of Li^+^ (0.76 Å) than that of Na^+^ (1.02 Å), the substitution of Na^+^ with Li^+^ in NaGdF_4_ nanocrystals can thus effectively modulate the crystal field, which is also supported by the higher binding energy of Li-F bond (577 kJ/mol) than that of Na-F bond (477 kJ/mol). Therefore, doping Li^+^ into NaGdF_4_:Yb,Er is favorable for enhancing both up- and down-conversion luminescence, similar to Zr^4+^ dopants.

### The Synergistic Crystal Field Modulation via Co-doping of Zr^4+^ and Li^+^

**Figure 6:**
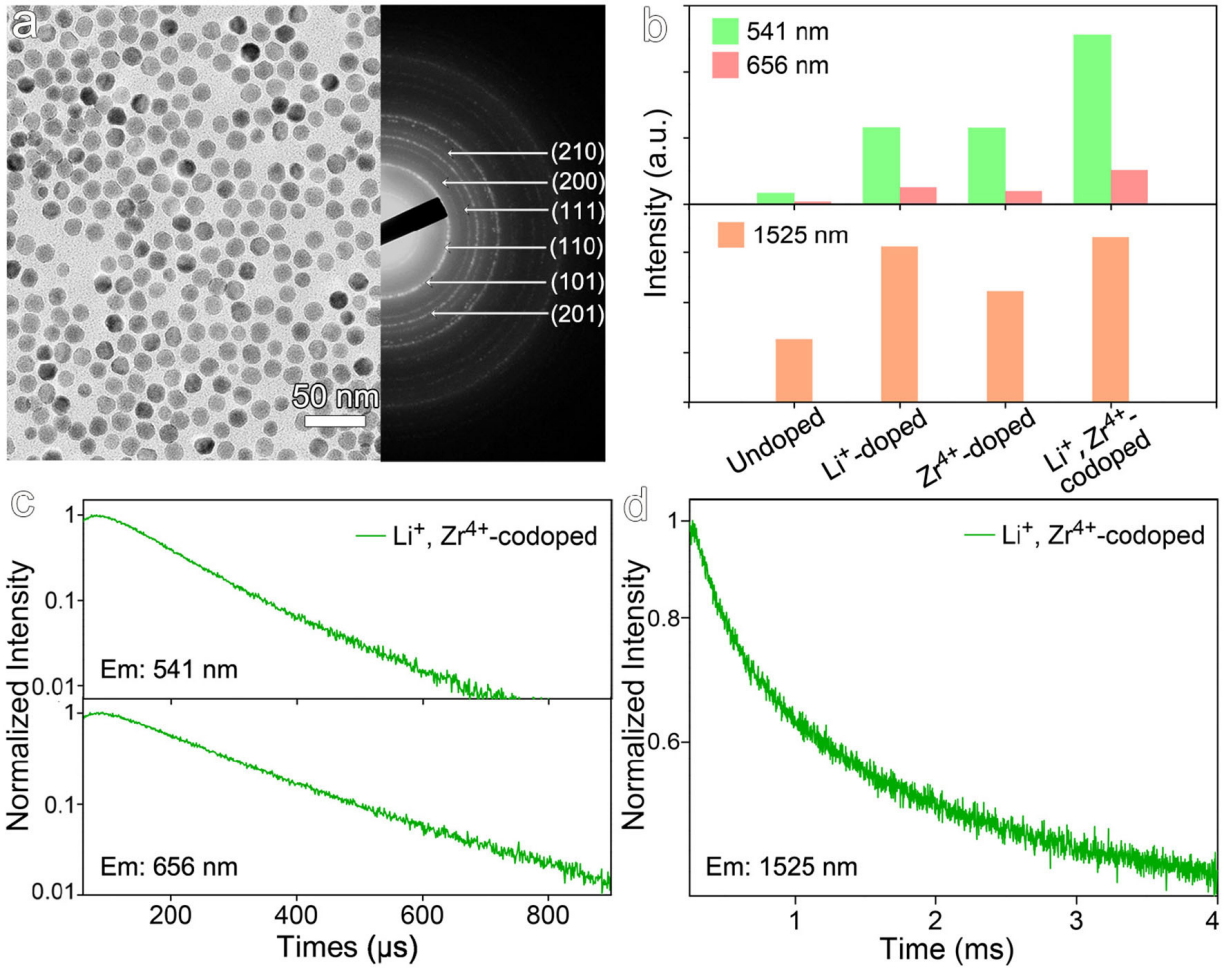
**(a)** TEM image and selected area electron diffraction pattern of NaGdF_4_:Yb,Er nanocrystals co-doped with Li^+^ and Zr^4+^. **(b)** The emission intensities at 541, 656, and 1,525 nm of NaGdF_4_:Yb,Er nanocrystals co-doped with Li^+^ and Zr^4+^ under 980 nm laser excitation. **(c,d)** Transient emissions observed at 541, 656, and 1,525 nm of NaGdF_4_:Yb,Er nanocrystals co-doped with Li^+^ and Zr^4+^ under 980 nm laser excitation.

Considering Zr^4+^ and Li^+^ ions occupy the different lattice sites, it's therefore very interesting to synergistically combine the effects of both Zr^4+^ and Li^+^ ions for further improving the optical properties of the NaGdF_4_:Yb,Er nanocrystals. The co-doped nanocrystals were then prepared. The TEM image and selected area electron diffraction pattern of the resulting nanocrystals shown in Figure 6a reveal that the average size is 15.8 ± 1.2 nm and the nanocrystals are in hexagonal phase. As shown in Figure 6b, upon co-doping of Zr^4+^ and Li^+^ ions, the luminescence intensity of up-conversion emission at 541 nm is enhanced by factors of 15.0, 2.2, and 2.2, if compared with those recorded from undoped, Li^+^-doped, and Zr^4+^-doped nanocrystals, respectively, while the down-conversion emission intensity of co-doped samples is enhanced by factors of 2.7, 1.1, and 1.5, respectively. As shown in Figures 6c,d and Table S5, the transient optical behaviors of the co-doped nanocrystals also confirm the synergistic effects of Zr^4+^ and Li^+^, evidenced by the prolonged average decay lifetimes for different emissions. For example, the decay lifetime of 541 nm emission is of 123.3 μs, higher than 82.1 μs for the undoped, 107.5 μs for Li^+^-doped, and 114.1 μs for Zr^4+^-doped ones.

## Conclusion

In summary, tetravalent Zr^4+^-doped NaGdF_4_:Yb,Er nanocrystals were prepared. The steady-state and transient luminescence spectroscopies studies demonstrated that the Zr^4+^ dopants could simultaneously enhance both up- and down-conversion luminescence of Er^3+^ ions. Combining the experimental results with Judd-Ofelt theoretical analysis, it was revealed that Zr^4+^ dopants take effects by varying the crystal field surrounding Er^3+^ emitters. Compared with host Gd^3+^ ion, Zr^4+^ ion exhibits increased electron cloud distortion ability due to its stronger electric polarizability and higher bond energy with F^−^, which accounts for the increased distortion of crystal field surrounding Er^3+^, thus efficiently enhancing both up- and down- conversion luminescence. In addition, the effects of non-Ln dopants (Sc^3+^, Mg^2+^, Mn^2+^) on the up- and down-conversion emission were also investigated, and no stronger effects than Zr^4+^ ion were observed. Apart from the substituting Gd^3+^ ion, strong luminescence enhancement effects were also found when Li^+^ ion was used to substitute Na^+^ via similar mechanism, which offers an opportunity to show the synergistic effects of Zr^4+^ and Li^+^ ions by co-doping the NaGdF_4_:Yb,Er nanocrystals with them. Upon optimization, the up-conversion emissions were enhanced by more than one order of magnitude compared with undoped nanocrystals. The current studies thus demonstrate that the local crystal field surrounding emitters is an effective parameter for manipulating the up- and down-conversion luminescence of lanthanide emitters.

## Materials and Methods

### Materials

GdCl_3_·6H_2_O (450855), YbCl_3_·6H_2_O (337927), ErCl_3_·6H_2_O (259256), ScCl_3_·6H_2_O (451274), oleic acid (OA, 364525), 1-octadecene (ODE, O806), oleylamine (OM, O7805), ammonium fluoride (NH_4_F, 216011) and potassium fluoride (KF, 402931) were all purchased from Sigma-Aldrich. LiOH·H_2_O (80074718), MgCl_2_·6H_2_O (10012817) and Mn(ac)_2_·4H_2_O (A22100702) were purchased from Sinopharm Chemical Reagent Beijing, Co., Ltd. Zr(acac)_4_ (Z107220) was purchased from Aladdin Chemistry Co., Ltd. Other analytical grade chemicals, such as cyclohexane, ethanol and sodium hydroxide, were purchased from Sinopharm Chemical Reagent Beijing, Co., Ltd.

### Synthesis of NaGdF_4_:Yb,Er Nanocrystals

The NaGdF_4_:Yb,Er nanocrystals were synthesized through the coprecipitation reaction at high temperature according to our previous works with slight modification (Liu et al., 2016). Typically, GdCl_3_·6H_2_O (0.48 mmol), YbCl_3_·6H_2_O (0.108 mmol), and ErCl_3_·6H_2_O (0.012 mmol) were mixed with 4 mL of OA as the coordinating ligand and 16 mL of ODE as the non-coordinating solvent in a 100 mL 3-necked flask. The mixture was heated to 150°C under a vacuum and then kept at this temperature approximately for 60 min for forming a homogeneous solution. 10 mL of methanol solution containing NaOH (1.8 mmol) and NH_4_F (1.8 mmol) was added dropwise after the solution was cooled down to 50°C. Then the reaction system was stirred at 50°C for another 30 min. After that, the methanol was removed under a vacuum and the solution was kept at 110°C for 10 min until there were no bubbles in the reaction system. Subsequently, the reaction temperature was improved to 300°C with the rate of 20°C/min, under a gentle flow of nitrogen protection. And the reaction was held for 1 h at 300°C. Finally, the reaction system was cooled to room temperature. The nanocrystals were precipated by ethanol, collected by centrifugation for several times and then re-dispersed in cyclohexane for further use.

### Synthesis of Non-ln Ions Doped NaGdF_4_:Yb,Er Nanocrystals

The rare-earth nanocrystals doped with different non-Ln metal ions were synthesized following a similar process of the preparation of NaGdF_4_:Yb,Er nanocrystals. In the case of Zr^4+^-doped NaGdF_4_:Yb,Er nanocrystals, different feeding ratios of Zr^4+^ ions (defined as the molar ratios of Zr^4+^ precursor to the amount of total cation precursors) were used, namely: 3.0, 5.0, 10.0, 15.0%. In detail, Zr(acac)_4_ (*x* mmol, *x* = 0.018, 0.03, 0.06, 0.09), GdCl_3_·6H_2_O (0.48-*x* mmol), YbCl_3_·6H_2_O (0.108 mmol), and ErCl_3_·6H_2_O (0.012 mmol) were mixed with 4 mL of OA and 16 mL of ODE in a 100 mL 3-necked flask. While the Sc^3+^, Mg^2+^ or Mn^2+^-doped NaGdF_4_:Yb,Er nanocrystals were synthesized similar to the Zr^4+^-doped nanocrystals (Zr^4+^ precursor:10.0%) by replacing Zr(acac)_4_ with ScCl_3_·6H_2_O, MgCl_2_·6H_2_O or Mn(ac)_2_·4H_2_O, respectively. In addition, for the Mn^2+^-doped NaGdF_4_:Yb,Er nanoparticles, the precursors were mixed with 3.8 mL of OA and 0.2 mL OM as the coordinating ligand. The growth of the nanocrystals and the following purification procedures were the same as those for the NaGdF_4_:Yb,Er nanocrystals.

In the case of Li^+^-doped NaGdF_4_:Yb,Er nanocrystals, after forming a homogeneous solution of rare-earth chloride at 50°C, 10 ml of methanol solution containing of NaOH (1.62 mmol), LiOH·H_2_O (0.18 mmol) and NH_4_F (1.8 mmol) was added in the reaction system for preparing Li^+^-doped NaGdF_4_:Yb,Er nanocrystals. Other experimental steps were the same to the synthesis of above-mentioned nanocrystals. While in the case of NaGdF_4_:Yb,Er nanoparticles doped with K^+^, the methanol solution was consisted of NaOH (1.62 mmol), KF (0.18 mmol) and NH_4_F (1.62 mmol). Other experimental steps were the same to the synthesis of above-mentioned nanoparticles.

### Synthesis of Li^+^ and Zr^4+^ Co-doped NaGdF_4_:Yb,Er Nanocrystals

GdCl_3_·6H_2_O (0.42 mmol), Zr(acac)_4_ (0.06 mmol), YbCl_3_·6H_2_O (0.108 mmol), and ErCl_3_·6H_2_O (0.012 mmol) were mixed with 4 mL of OA and 16 mL of ODE. After forming a homogeneous solution of rare-earth chloride at 50°C, 10 ml of methanol solution containing of NaOH (1.62 mmol), LiOH·H_2_O (0.18 mmol) and NH_4_F (1.8 mmol) was added in the reaction system for preparing Li^+^ and Zr^4+^ co-doped NaGdF_4_:Yb,Er nanocrystals. Other experimental steps were the same to the synthesis of above-mentioned nanocrystals.

### Structural and Optical Characterization of Nanocrystals

Transmission electron microscopy (TEM) measurements were carried out with JEM-100CXII at 100 KV and Hitachi HT7700 at 120 KV for characterizing the particle shape and size, and selected area electron diffractions (SAED) were taken for the characterization of crystalline structure. The particle size was determined with ImageJ by counting more than 300 nanoparticles per sample. In addition, X-ray diffraction (XRD) measurements were carried out on a Regaku D/Max-2500 diffractometer under Cu Kα_1_ radiation (λ = 1.54056 Å) for further characterizing the phase structure of the resultant nanocrystals. The concentrations of metal ions in different systems was determined by using an inductively coupled plasma atomic emission spectrometer (ICP-AES 6300DV) after the particles were eroded with certain concentrated mixture of concentrated nitric acid and hydrogen peroxide. The up- and down-conversion luminescence spectra were measured by a Cary Eclipse fluorescence spectrophotometer and Edinburgh Instruments FLS 920 equipped with 980 nm CW laser diodes (MDL-III-980 nm) as the excitation sources, respectively. The luminescence spectra of different samples are normalized according to the concentration of Er^3+^ serving as the emissive centers, and the excitation power (2 W/cm^2^) remain unchanged for comparing the luminescence intensities of different samples dispersed in cyclohexane. The transient up- and down-conversion luminescence of different samples dispersed in cyclohexane was recorded on FLS 920 equipped with 980 nm pulsed laser diodes (the excitation power: 2 W/cm^2^, pulse frequency: 100 Hz, pulse width: 3 μs), with the former detected by HAMAMATSU PMT (R928-P) detector and the latter detected by HAMAMATSU NIRPMT (R5509) detector.

## Data Availability Statement

All datasets generated for this study are included in the article/Supplementary Material.

## Author Contributions

MG, CL, LJ, and YL were responsible for designing experiments, put forward the innovation of this work, and wrote the paper. YL carried out the experiments and analyzed the data with the support from MG, LJ, and CL. All the authors contributed to the discussion of results and the design of figures.

## Conflict of Interest

The authors declare that the research was conducted in the absence of any commercial or financial relationships that could be construed as a potential conflict of interest.
