# Supplementary material for: Doping Lanthanide Nanocrystals With Non-lanthanide Ions to Simultaneously Enhance Up- and Down-Conversion Luminescence
Source: Front Chem. 2020 Sep 23;8:832. doi: 10.3389/fchem.2020.00832 (PMC7538674; doi:10.3389/fchem.2020.00832)
Supplement: Supplementary file 1 [file Data_Sheet_1.pdf]

Supplementary Material

Doping Lanthanide Nanocrystals with Non-lanthanide ions to Simultaneously Enhance Up- and Down-conversion Luminescence

Yingying Li,[1,2] Chunyan Liu,[1*] Peisen Zhang,[1,2] Jiayi Huang,[1,2] Haoran Ning,[1,2] Peng Xiao,[1,2] Yi Hou,[1] Lihong Jing,[1*] Mingyuan Gao[1,2,3*]

[1]Key Laboratory of Colloid, Interface and Chemical Thermodynamics, Institute of Chemistry, Chinese Academy of Sciences, Bei Yi Jie 2, Zhong Guan Cun, Beijing 100190, P. R. China

[2]School of Chemistry and Chemical Engineering, University of Chinese Academy of Sciences, Beijing 100049, P. R. China

[3]Center for Molecular Imaging and Nuclear Medicine, School for Radiological and Interdisciplinary Sciences (RAD-X), Collaborative Innovation Center of Radiation Medicine of Jiangsu Higher Education Institutions, and State Key Laboratory of Radiation Medicine and Protection, Soochow University, Suzhou 215123, China.

*** Correspondence:**
gaomy@iccas.ac.cn, jinglh@iccas.ac.cn, liuchy@iccas.ac.cn.

Table S1 The ICP-AES results of cation ratio in Zr^{4+}-doped $NaGdF_4$:Yb,Er nanocrystals.

Feeding molar ratio of Zr^{4+} (%)	Concentrations of different ions (mmol/L)				Amount of Gd^{3+} (%)
	Gd^{3+}	Yb^{3+}	Er^{3+}	Zr^{4+}	
0	2.93	0.63	0.10	0	80.1
3	4.89	1.06	0.13	0.15	78.5
5	3.56	0.85	0.10	0.17	76.1
10	4.17	0.93	0.12	0.40	74.2
15	3.36	0.85	0.09	0.47	70.4

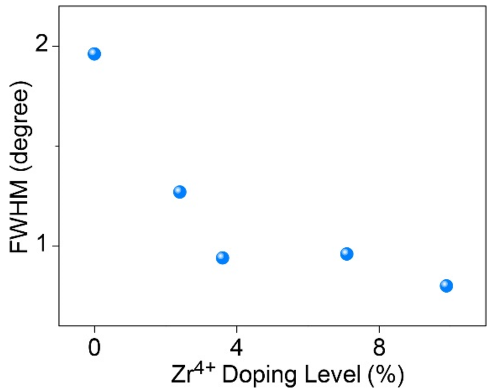

Figure S1 The full width at half maximum of diffraction peak for (201) crystal plane of Zr^{4+}-doped $NaGdF_4$:Yb,Er nanocrystals as a function of Zr^{4+} doping level.

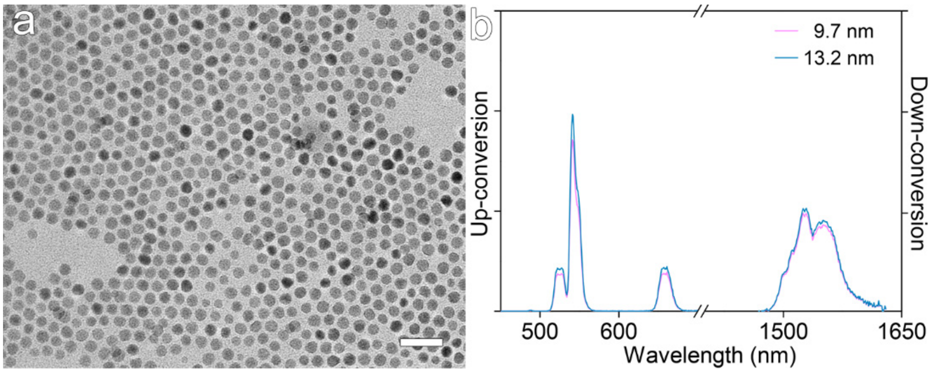

Figure S2 (a) TEM image of the 13.2 nm sized $NaGdF_4$:Yb,Er nanocrystals. The scale bar embedded corresponds to 50 nm. (b) Up- and down-conversion luminescence spectra of different sized $NaGdF_4$:Yb,Er nanocrystals under 980 nm laser excitation.

Methods for Photoluminescence Lifetime Fitting

The photoluminescence decay curves are fitted by using bi-exponential function (Eq. S1)

$$I(t) = \sum_{i=1}^{n} B_i \exp(-t/\tau_i), \qquad \sum_{i=1}^{n} B_i = 1 \qquad \text{(S1)}$$

In this expression, τ_i represents the decay time constants, and B_i represents the normalized amplitudes of each components, n is the number of decay times.

The amplitude is calculated by using Eq. S2.

$$\tau_{avg} = (\sum_{i=1}^{n} B_i \tau_i^2)/(\sum_{i=1}^{n} B_i \tau_i) \qquad \text{(S2)}$$

Table S2 The two-exponential fitting of luminescence decay curves at 541 nm, 656 nm, and 1525 nm of Zr^{4+}-doped $NaGdF_4$:Yb,Er nanocrystals upon excitation of 980 nm.

EX=980 nm	541 nm					656 nm				
	τ_1 (μs)	B_1 (%)	τ_2 (μs)	B_2 (%)	τ_{avg} (μs)	τ_1 (μs)	B_1 (%)	τ_2 (μs)	B_2 (%)	τ_{avg} (μs)
0% Zr^{4+}	65.9	76	112.0	24	82.1	81.0	93	197.3	7	98.7
2.4% Zr^{4+}	82.4	80	154.2	20	104.8	104.8	90	364.9	10	177.9
3.6% Zr^{4+}	92.9	97	265.2	3	108.5	100	93	249.3	7	124.5
7.1% Zr^{4+}	94.9	96	266.6	4	114.1	101.6	91	286.5	9	141.9
9.9% Zr^{4+}	92.8	92	213.8	8	113.4	101	92	296.8	8	140.9

EX=980 nm	1525 nm				
	τ_1 (μs)	B_1 (%)	τ_2 (μs)	B_2 (%)	τ_{avg} (μs)
0% Zr^{4+}	236.8	43	968.4	57	855.0
2.4% Zr^{4+}	241.6	39	1210.2	61	1100.6
3.6% Zr^{4+}	244.6	47	1398.8	53	1243.8
7.1% Zr^{4+}	272.2	41	1379.7	59	1247.2
9.9% Zr^{4+}	284.6	43	1501.4	57	1347.5

Table S3 The two-exponential fitting of luminescence decay curves at 541 nm, 656 nm, and 1525 nm of $NaGdF_4$:Yb,Er nanocrystals doped with cations of lower valance state, e.g., Sc^{3+}, Mg^{2+} upon excitation of 980 nm.

EX=980 nm	541 nm					656 nm				
	τ_1 (μs)	B_1 (%)	τ_2 (μs)	B_2 (%)	τ_{avg} (μs)	τ_1 (μs)	B_1 (%)	τ_2 (μs)	B_2 (%)	τ_{avg} (μs)
Sc^{3+}-doped	68.9	84	131.6	16	85.9	100.5	95	250.1	5	118.1
Mg^{2+}-doped	62.4	73	122.1	27	87.4	87.0	89	208.5	11	115.4

EX=980 nm	1525 nm				
	τ_1 (μs)	B_1 (%)	τ_2 (μs)	B_2 (%)	τ_{avg} (μs)
Sc^{3+}-doped	239.3	41	1153.2	59	1035.9
Mg^{2+}-doped	201.1	43	1070.8	57	962.8

Table S4 The two-exponential fitting of luminescence decay curves at 541 nm, 656 nm, and 1525 nm of Li^{+}-doped $NaGdF_4$:Yb,Er nanocrystals upon excitation of 980 nm.

EX=980 nm	541 nm					656 nm				
	τ_1 (μs)	B_1 (%)	τ_2 (μs)	B_2 (%)	τ_{avg} (μs)	τ_1 (μs)	B_1 (%)	τ_2 (μs)	B_2 (%)	τ_{avg} (μs)
Li^{+}-doped	96.7	97	258.2	3	107.5	124.9	96	365.1	4	151.1

EX=980 nm	1525 nm				
	τ_1 (μs)	B_1 (%)	τ_2 (μs)	B_2 (%)	τ_{avg} (μs)
Li^{+}-doped	280.2	39	1425.8	61	1297.5

Table S5 The two-exponential fitting of luminescence decay curves at 541 nm, 656 nm, and 1525 nm of $NaGdF_4$:Yb,Er nanocrystals co-doped with Li^{+} and Zr^{4+} upon excitation of 980 nm.

EX=980 nm	541 nm					656 nm				
	τ_1 (μs)	B_1 (%)	τ_2 (μs)	B_2 (%)	τ_{avg} (μs)	τ_1 (μs)	B_1 (%)	τ_2 (μs)	B_2 (%)	τ_{avg} (μs)
Li^{+}, Zr^{4+}-codoped	110.9	98	374.8	2	123.3	149.2	87	305.2	13	184.7

EX=980 nm	1525 nm				
	τ_1 (μs)	B_1 (%)	τ_2 (μs)	B_2 (%)	τ_{avg} (μs)
Li^{+}, Zr^{4+}-codoped	276.5	46	1559.2	54	1388.1
